# Ketamine Infusion Used to Successfully Control Refractory Status Epilepticus in a Pregnant Patient

**DOI:** 10.1155/2018/3041279

**Published:** 2018-10-25

**Authors:** Murad Talahma, Vivek Sabharwal, Yana Bukovskaya, Fawad Khan

**Affiliations:** Department of Neurocritical Care, Ochsner Health System, New Orleans, LA, USA

## Abstract

The management of SE during pregnancy is especially challenging to the treating physician. While antiepileptic medications might cause teratogenicity, SE can have significant morbidity and mortality on both the mother and the developing fetus. This case report demonstrated the successful use of ketamine infusion in the management of RSE in pregnancy without affecting the immediate outcome of pregnancy. The fetus survived this complicated ICU stay and outpatient follow-up was generally uncomplicated. The pregnancy was ended with a delivery of a normal female newborn.

## 1. Introduction 

Worldwide, an estimated half million women of child-bearing age have been diagnosed with epilepsy [1]. The management of seizures during pregnancy poses the challenge of striking a balance between the risks of complications from seizures in the mother and the possible teratogenic effects of antiepileptic drugs (AEDs) on the developing fetus. Ketamine is an N-methyl-D-aspartate (NMDA) receptor antagonist that has been used with increasing frequency in the management of refractory and super-refractory status epilepticus (SE); however, to our knowledge, its successful use in pregnancy has never been reported. We present a case in which ketamine was used to manage refractory SE in a pregnant patient without affecting the outcome of the pregnancy.

## 2. Case Report

The patient is a 37-year-old female with a history of epilepsy secondary to astrocytoma that had been surgically resected and followed with radiation and chemotherapy a year prior to the current presentation. Her seizure semiology ranged from focal seizures to generalized tonic-clonic seizures. Her outpatient AEDs included levetiracetam (LEV), valproic acid (VPA), and zonisamide (ZNS). Ten days prior to her presentation, she discovered that she was pregnant and decided to self-discontinue her VPA. She experienced a significant increase in her seizure frequency, for which she was admitted to our neurocritical care unit (NCCU). Initially her home doses of LEV and ZNS were increased from 1500 mg bid to 2000 mg bid and from 200 mg bid to 300 mg bid, respectively. The patient's blood levels of LEV and ZNS on admission were 23 ug/mL and 29 mcg/mL, respectively, which are within therapeutic ranges.

On day 2 of her hospitalization, she was started on daily prenatal vitamins in addition to 4 mg folic acid. Transvaginal ultrasound showed a single intrauterine pregnancy corresponding with a 6-week, 6-day gestation by crown rump length.

The patient continued to have intermittent seizures involving both sides of the face with associated confusion. She was placed on continuous electroencephalogram (EEG) monitoring that showed right hemisphere focal SE. Her seizures continued at a rate of multiple episodes per hour, and she failed to respond to a total of 10 mg of lorazepam administered in 2 mg doses; thus the decision was made to intubate and start anesthetic agents. Continuous propofol infusion was initiated without a bolus dose at a rate of 30 mcg/kg/min and titrated to 45 mcg/kg/min; however, further up titrations were not tolerated because of dose related hypotension. She received a bolus of 80 mg ketamine intravenously (IV) and was started on a continuous ketamine infusion at a rate of 100 mcg/kg/min. Additionally, a continuous infusion of magnesium sulfate was initiated. Her EEG continued to show right focal SE presenting with both clinical and subclinical seizures; thus the ketamine infusion rate was increased to 150 mcg/kg/min. Nine hours after the initiation of ketamine, the seizures stopped both clinically and electrographically. Twenty-four hours later, propofol was discontinued. Twenty-four hours after propofol was stopped, the seizure suppression continued, so the ketamine infusion was gradually decreased to 75 mcg/kg/min while continuing her maintenance AED regimen, which included LEV 3000 mg q8h, ZNS 300 mg bid, oxcarbazepine 300 mg bid, and lacosamide 200 mg bid. On day 7 of ketamine, the infusion rate was decreased to 50 mcg/kg/min for 6 hours, then to 30 mcg/kg/min, and subsequently discontinued. The patient remained seizure-free both clinically and electrographically (Table 1).

She remained intubated for a total of 8 days and was successfully extubated. She remained on EEG monitoring for 3 additional days which showed no seizures. After 2 weeks in the NCCU, she was transitioned to a regular floor. On day 18 of her hospitalization, ZNS was discontinued. She remained in the hospital for an additional 5 days, experienced no clinical seizures, and was subsequently discharged home.

Multiple ultrasounds after discharge showed a normal fetus, appropriate to gestational age, and normal amniotic fluids. Fetal echocardiogram showed no evidence of cardiac anomalies. The patient was admitted for elective caesarean section at 37 weeks and 5 days' gestation and delivered a single viable female. The baby scored 9 on both the 1-minute and 5-minute Apgar scores. The patient and the newborn were discharged 4 days postoperatively in stable condition. At the most recent follow-up visit 38 weeks after the birth of the baby discharge she denied any further episodes of status epilepticus. She reported no cognitive deficits or mood changes. Her baby was brought to the clinic and was notably healthy while achieving all normal developmental milestones.

## 3. Discussion

The definition of SE has evolved over the years, starting with the initial definition in 1993 by the Epilepsy Foundation of America that required repetitive seizures for 30 minutes; however, the time was subsequently reduced to 20, then 10, and most recently 5 minutes [2]. When SE fails to respond to 2 AEDs, it is referred to as refractory SE. When seizure activity continues for 24 hours or more after initiation of anesthetic treatment, it is considered super-refractory SE [3]. An estimated 31%-43% of patients with SE will develop refractory SE [4] and 10%-15% will develop super-refractory SE [3]. Both refractory and super-refractory SE carry a significantly higher mortality and morbidity than SE, and their management poses a significant challenge, particularly given the absence of strong evidence or consensus in the literature to guide management.

The relationship between pregnancy and seizures is complicated, given the physiological changes of pregnancy on seizure control and AED requirements. Seizures are not only harmful to the mother, but hypoxia and acidosis resulting from convulsive seizures can have a harmful effect on the developing fetus as well. Although most women with epilepsy will be seizure-free during pregnancy, approximately one-third will have an increase in their seizure frequency [5], and the timing of the seizures is evenly distributed among trimesters [6]. The frequency of SE in pregnancy ranges from 0% to 1.3% compared to 1.6% in the general epilepsy population [1]. Compliance with AEDs might be a major factor in the control of seizures during pregnancy, but other factors such as hormonal changes, changes in protein binding that affect the volume of distribution of AEDs, and the change in renal clearance and intestinal absorption of AEDs may affect seizure control and management during pregnancy.

One of the primary factors in the development of both refractory and super-refractory SE is the unique changes in the neuroreceptors at the synapses and blood brain barrier. As seizures continue, the GABA A receptors move to the inner side of the membrane, while the NMDA receptors move in the opposite direction [2]. These changes explain the development of pharmacoresistance to the AEDs, such as benzodiazepines and propofol, that work on GABA receptors as the seizure activity continues. The finding that NMDA receptors are upregulated during SE increased interest in using agents such as ketamine that block NMDA receptors. The neurocritical care community has been reluctant to use ketamine to treat SE patients because of reports in the literature from the 1960s and 1970s that showed the potential for ketamine to increase the intracranial pressure in neurologically ill patients; however those reports have been contradicted by more recent data from patients with both traumatic and nontraumatic brain injury [7].

Conflicting data about the safety of ketamine has been published. While early studies showed that ketamine can be neuroprotective [8], others showed that ketamine can cause cerebral atrophy [9]. Similar conflicting results have been reported in animal studies. In one study of rats with traumatic brain injury, ketamine was associated with neuronal apoptosis [7], while others showed that ketamine can prevent learning impairment when administered immediately after the onset of SE [10].

Ketamine use in the management of SE provides a promising choice given its unique mechanism of action on the NMDA receptors, availability, fast onset of action, and generally benign side effect profile. Some of the side effects of ketamine include hallucinations, arrhythmia, and hypersalivation. The sympathetic properties of ketamine might be helpful if hypotension is a concern, but ketamine should be used with caution or avoided in patients with coronary artery disease or severe hypertension.

Reports about the use of ketamine during pregnancy in the literature are primarily cases of anesthesia for caesarian section, with some reports of recreational use of the drug during pregnancy. Some animal studies have shown teratogenic effects of ketamine. Ketamine crossed the placenta rapidly and equilibrated between the maternal and fetal circulation when studied in pregnant ewes [11]. The litter pregnant rats that received ketamine in their second trimester had memory impairment, as well as depression-like and anxiety-like behavioral disorder [12]. A report of an infant's late exposure to recreational ketamine showed some association with intrauterine growth retardation, remarkable hypotonia, and poor reflexes [13].

To our knowledge, this is the first case report in the literature of ketamine use for refractory SE during pregnancy. Our patient experienced resolution of RSE with the use of intravenous infusion of ketamine. She remained on ketamine infusion for a total of 7 days. No acute adverse effects were noted. She reported no long-term adverse effects after discharge from the hospital. No complications occurred during the caesarean Section. At 9 months the baby was healthy and achieved all development milestones.

## 4. Conclusion

The management of SE during pregnancy is especially challenging to the treating physician. While antiepileptic medications might cause teratogenicity, SE can have significant morbidity and mortality on both the mother and the developing fetus. This case report demonstrated the successful use of ketamine infusion in the management of RSE in pregnancy without affecting the immediate outcome of pregnancy. The fetus survived this complicated ICU stay and outpatient follow-up was generally uncomplicated. The pregnancy was ended with a delivery of a normal female newborn.

## Figures and Tables

**Table 1:** “The timeline of different antiepileptic medications during ICU stay”.

Therapy in ICU [Day 1-Day 15]			
Anesthetics			

		Dose Range	Therapeutic Monitoring

Ketamine	Day 3-9	50 -150 mcg/kg/min	N/A

Propofol	Day 3-5	10-40 mcg/kg/min	N/A

Maintenance AEDs			

		Total Daily Dose	

Levetiracetam	Day 1-15	3000mg-9000mg	18.4-88.9 ug/mL

Lacosamide	Day 2-15	400 mg-500 mg	

Oxcarbazepine	Day 4-15	600 mg-800 mg	5-10 mcg/mL

Zonisamide	Day 7-15	600 mg	11-29 mcg/mL

Ancillary Therapy			

Pyridoxine	Day 3-15	50 mg	3 ug/L

Magnesium Sulfate Infusion	Day 3-7	Maintain serum Mg 3-3.5	3.1-3.8 mg/dL

IV Methylprednisolone	Day 4-6	1000 mg	N/A
